# Multidimensional regulatory roles and therapeutic applications of SRSF7 in cancer

**DOI:** 10.1038/s41420-025-02937-4

**Published:** 2025-12-30

**Authors:** Yuan Li, Huimeng Gao, Xuanyu Zhang, Fuli Sun, Yan Guo, Xue Qiao

**Affiliations:** 1 Department of Oral Biology, School and Hospital of Stomatology, China Medical University, Liaoning Province Key Laboratory of Oral Disease, Shenyang, Liaoning 110002 China; 2 Department of Central Laboratory, School and Hospital of Stomatology, China Medical University, Liaoning Province Key Laboratory of Oral Disease, Shenyang, Liaoning 110002 China

**Keywords:** Cell polarity, Cancer epidemiology

## Abstract

Malignant tumors, as one of the leading causes of mortality, pose great threats to global public health. Serine/Arginine-rich Splicing Factor 7 (SRSF7), a core splicing regulatory protein of the SRSF family, plays a crucial role in maintaining RNA stability, facilitating alternative splicing, and assisting RNA nuclear export. It also exhibits significantly aberrant expression among various cancers, including lung, colorectal, liver, and oral cancer. This review examines the molecular mechanisms of SRSF7 in tumorigenesis, with a focus on its role in the epigenetic reprogramming of related tumors. Specifically, it explores the abnormal regulation of the cell cycle, the regulation of non-coding RNA, the control of RNA methylation, and the reprogramming of glucose metabolism. Additionally, this review examines the role of SRSF7 in the tumor immune microenvironment through alternative splicing and immune evasion through the immune checkpoint PD-1. It also highlights the role of SRSF family members in tumor resistance, illustrating how alternative splicing contributes to tumor chemoresistance. Although SRSF7 shows significant promise in tumor intervention therapies, more experimental and clinical studies are still needed to evaluate its clinical application. This review enhances our understanding of the molecular landscape of SRSF7 in tumorigenesis with great potential to become a key node in tumor-targeted therapy and companion diagnostics, driving translational potential from mechanisms to clinical applications.

## Facts

- SRSF7 is abnormally expressed in various malignant tumors, including lung, colorectal, liver, and oral cancers.
- SRSF7 promotes tumor development through epigenetic reprogramming mechanisms, such as regulating the cell cycle, non-coding RNAs, RNA methylation, and glucose metabolism.
- SRSF7 can influence the tumor immune microenvironment and immune evasion by affecting IRF7 expression or the alternative splicing of immune checkpoints like PD-1.
- Alternative splicing regulated by SRSF family members is a crucial factor in tumor chemoresistance.

## Open Questions

- The detailed molecular networks by which SRSF7 causes abnormal splicing events and influences downstream gene expression in tumors are still unclear. How does it specifically coordinate epigenetic reprogramming?
- Besides PD-1 and IRF7, what other important immune molecules could be SRSF7’s targets through alternative splicing to affect anti-tumor immune responses within the tumor immune microenvironment?
- The regulatory mechanisms responsible for the often observed increase in transcription and translation of SRSF7 in most tumors have not been fully explained. What are the main factors involved?

## Introduction

According to the World Health Organization, cancer represents the second leading cause of mortality worldwide and constitutes one of the principal health challenges confronting humanity [1, 2]. Early-stage malignancies are often prone to misdiagnosis and missed diagnosis, which significantly affects the prognosis and survival of cancer patients. Alternative splicing (AS) is a vital process in eukaryotic gene regulation. It produces various mature mRNA variants by selectively splicing exons and removing introns from pre-mRNA. These isoforms can be translated into protein variants that differ in structure or function, thereby significantly increasing the diversity of the functional proteome [3–5]. The dysregulation of alternative splicing is increasingly recognized as a crucial mechanism in malignancy. It can disrupt the equilibrium between oncogenes and tumor suppressor genes, thereby affecting various aspects of carcinogenesis, including invasion, immune response, and drug sensitivity [6–8]. Therefore, it is essential to identify tumor-specific biomarkers that regulate the alternative splicing process to assist in early tumor diagnosis or enhance precision treatment.

In the area of alternative splicing, the family of serine/arginine-rich splicing factors (SRSFs) has been extensively studied. This family includes 12 members, from SRSF1 to SRSF12, which typically regulate gene expression and cell cycle distribution through splicing variations, promoting carcinogenesis [9]. Among them, the serine/arginine-rich splicing factor 7 (SRSF7) is primarily responsible for splicing large protein complexes and is widely involved in regulating RNA splicing, immunoregulation, cell cycle regulation, and maintaining stem cell characteristics [10, 11]. Researches indicate that SRSF7 is abnormally over-expressed in various cancers, including colon cancer, liver cancer, oral cancer, and non-small cell lung cancer [12–15]. Pan-cancer analysis reveals that SRSF7 is upregulated and closely correlated with overall survival (OS) in cancers and knockdown SRSF7 leads to apoptosis of osteosarcoma cells [16]. SRSF7 was also recognized as a member of the AS-signature prognostic models and was significantly related to OS, immune cells infiltration, and cancer-related pathways in STAD [17]. SRSF7 not only regulates cell proliferation, apoptosis, migration, and invasion, but also influences immune cells recruiting to modulate the tumor micro-environment [18]. However, the specific mechanisms by which SRSF7 recognizes and participates in tumor-specific splicing variations still require further in-depth investigation.

This review aims to comprehensively elucidate the complex regulatory mechanisms of SRSF7 in tumor biology and its therapeutic applications, based on its biological functions and its role in carcinogenesis, to provide a theoretical foundation for further exploration of the clinical treatment potential of SRSF7.

## Molecular structure and biological roles of SRSF7

### Basic molecular structural characteristics of SRSF7

The SRSF family consists of 12 members, all of which are highly conserved in structure. The carboxyl terminus (C-terminal) of the proteins typically contains one or two RNA recognition motif (RRM) domains. The RRM domain, an essential RNA-binding region, can interact with both precursor mRNAs and messenger RNAs (mRNAs). The differences in this region determine how various SRSF family members recognize distinct precursor mRNAs and RNA substrates. The amino terminus (N-terminal) of the SRSF proteins contains one or more Arginine-Serine rich domains (RS), which serve as sites for protein-protein interactions and are also major sites for post-translational modifications [18, 19].

**Fig. 1:**
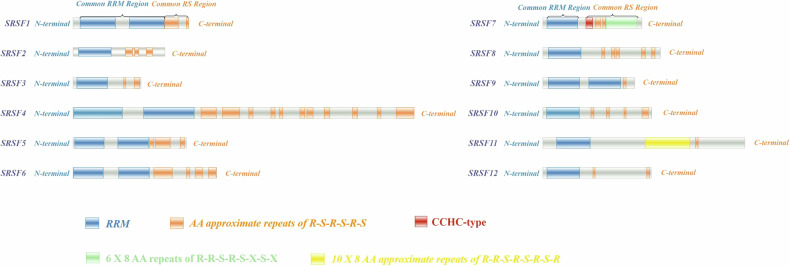
Structural organization of serine/arginine-rich splicing factor (SRSF) family members. The SRSF family primarily consists of one or two RRM domains at the N-terminus and a RS domain at the C-terminus. Notably, SRSF7 contains a CCHC-type zinc knuckle domain. Structural information was retrieved from UniProt (https://www.uniprot.org/). AA amino acid, N-terminus amino terminus, C-terminus carboxyl terminus, CCHC-type Cys-Cys-His-Cys type, RRM RNA recognition motif, RS serine/arginine-rich.

The spliceosome is essentially a multi-subunit RNA/protein complex composed of five small nuclear RNAs (U1, U2, U4, U5, and U6) and numerous small nuclear ribonucleoproteins (snRNPs). During assembly and catalysis, it undergoes multiple rearrangements to precisely identify splice sites and catalyze the removal of introns and the joining of exons, ultimately completing the mRNA splicing process [20]. The SRSF family facilitates spliceosome assembly and activation by recruiting various small nuclear ribonucleoproteins [21, 22]. SRSF7, also known as 9G8, is a classic member of the SR protein family. It encodes both the RRM and SR domains and is the only SR family protein that encodes a C-X(2)-C-X(4)-H-X(4)-C (CCHC) zinc finger domain. This structure is believed to facilitate the specific binding of SRSF7, enabling it to bind to single-stranded nucleic acids, particularly single-stranded RNA (Fig. 1) [23–25].

### Major biological functions of SRSF7

#### Classic splicing function of SRSF7

Alternative splicing allows a gene to produce multiple mRNA isoforms with different structures and functions. Typically, alternative splicing consists of seven types: exon skipping (ES), alternative 5′splice site (A5SS), alternative 3′splice site (A3SS), mutually exclusive exons (MXE), intron retention (IR), alternative promoter usage (APS), and alternative polyadenylation (APA) [26]. SR proteins and heterogeneous nuclear ribonucleoproteins (hnRNPs) are the two prominent RNA-binding protein families involved in the splicing process [27].

In the early stages of splicing, the U1 small nuclear RNA and protein complex (U1 snRNP) recognizes and binds to the 5′splice site of the precursor mRNA. The splicing factor SF1 (Splicing Factor 1) and the auxiliary splicing factor U2AF65 (U2 small nuclear RNA auxiliary factor 65 kDa) bind to the branch point sequence (BPS) at the 3’ end of the intron and the polypyrimidine tract (PPT), respectively, forming an essential early intermediate E complex. Then, U2 snRNP pairs with the BPS, replacing SF1, to create the A complex. The recruitment of the U4/U6/U5 tri-snRNP complex forms the B complex. Subsequently, the U5 snRNP binds to the 3’-splice site, while the U6 snRNP binds to the U2 snRNP. Finally, through two ester exchange steps, the intron folds into a lariat structure, and the two exons are joined together, completing the alternative splicing process [28]. Current studies have reported that SR family proteins can assist in splicing by binding to exonic splicing enhancers (ESEs), recruiting and stabilizing splicing factors (such as U1 snRNP and U2AF) to help assemble and activate the spliceosome, thereby participating in most steps of alternative splicing [4]. SRSF7 primarily interacts with splicing factors during the splicing reaction through its SR domain, influencing splice site selection and regulating gene expression via alternative splicing.

#### Non-classical splicing function expansion of SRSF7

##### Involvement in nuclear export

mRNA nuclear export transfers mRNA from the nucleus to the cytoplasm, mainly via nuclear export factors like NXF1. This process ensures that properly processed mRNA leaves the nucleus to reach the cytoplasm, where it undergoes transcription and translation, completing the gene expression process. Additionally, the regulation of nuclear export also relies on RNA-binding proteins that recognize and handle mRNA [29]. The SRSF family participates to varying degrees in interactions with nuclear export factors like NXF1, thereby influencing the nuclear export process [30]. Among them, SRSF7 acts as an adaptor protein by binding to the last exon and the 3’ untranslated region (3′ UTR) of mRNAs. It recruits the nuclear export factor NXF1 to specific regions of the mRNAs, thus promoting the nuclear export process [30–32]. The transcription-export complex and TAP pathway (TREX-TAP) is a key RNA export pathway responsible for transporting mature mRNAs from the nucleus to the cytoplasm. This pathway is crucial for gene expression within the cell, guaranteeing that processed and spliced mRNAs are safely exported to the cytoplasm for translation [33]. However, the conventional nuclear export pathway is less effective at regulating long non-coding RNAs (lncRNAs) without introns in the cytoplasm. These lncRNAs, due to the lack of splicing, cannot automatically recruit the TREX complex, unlike lncRNAs with introns. Recent studies have shown that in the lncRNA NKILA, which lacks introns, a cytoplasmic accumulation region (CAR-N) exists. SRSF1 and SRSF7 localize to CAR-N, where they interact with key components of the TREX-TAP pathway, namely UAP56 and ALYREF. This interaction triggers the recruitment of the nuclear export factor NXF1, thereby completing the nuclear export process, blocking IkB phosphorylation and suppressing breast cancer [34, 35].

##### Participate in transcription and translation regulation

In addition to participating in nuclear export, SRSF7 also regulates mRNA transcription and translation. Research by Zhao et al. showed that during influenza virus infection in humans, SRSF7 can inhibit the polymerase activity of the PB2627E protein of the influenza A virus. The degree of inhibition is closely related to the length of the C-terminal RS domain [36]. Research by Oliver et al. found that SRSF7 could recruit FIP1 upstream of the pPAS and activate pPAS activity through interactions between its RS domain and FIP1. This recruitment shortens the 3’ UTR length during transcription, enhances RNA stability, increases the efficiency of protein translation, and promotes protein expression [37].

Nonsense-mediated mRNA decay (NMD) is a cellular quality control mechanism that degrades mRNA with premature termination codons (PTCs), preventing harmful truncated proteins [38]. Studies have shown that the over-expression of SRSF7 can promote the generation of transcripts containing pervasive cytotoxic exons (PCE), which contain premature termination codons and therefore trigger the NMD pathway [39, 40]. However, other studies suggest that the over-expression of SRSF7 can also inhibit NMD, allowing these PCE transcripts to be translated. Through split open reading frames (Split-ORFs), the PCE transcripts are translated into two truncated proteins, thereby regulating the maintenance of their protein homeostasis [41].

**Fig. 2:**
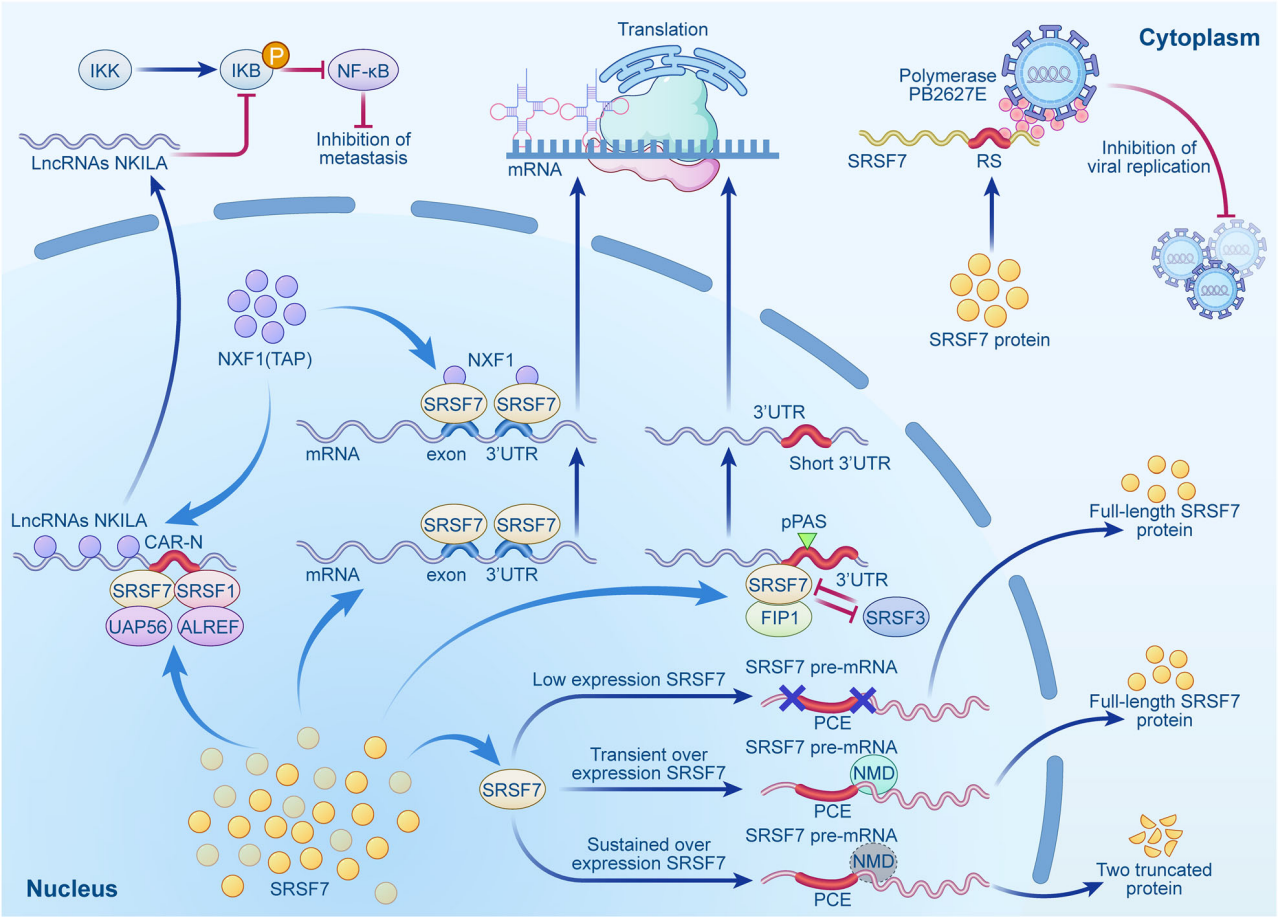
Non-canonical splicing functions of SRSF7. SRSF7 regulates gene expression through multiple mechanisms: (1) It facilitates the nucleocytoplasmic export of RNA by directly or indirectly recruiting the NXF1 through binding to mRNA termini or to the CAR-N of the long non-coding RNA NKILA, thereby participating in the TREX complex-TAP pathway or orchestrating the recruitment of the TREX complex, and further engaging in downstream pathways such as IκB phosphorylation suppression; (2) SRSF7 inhibits the polymerase activity of influenza A virus PB2-627E via its RS domain, interacting with FIP1 to activate pPAS, shortening the 3’UTR and enhancing RNA stability/translation efficiency; (3) SRSF7 overexpression exerts a dual regulatory role on transcripts containing PCEs: it not only promotes their production and triggers NMD but can also suppress NMD under specific conditions, enabling the translation of these transcripts via Split-ORFs to produce functional truncated proteins, thereby fine-tuning its own proteostasis. 3’UTR 3’Untranslated Region, ALREF Aly/REF-Dependent Recruitment Complex for Export; CAR-N Cytoplasmic Accumulation Region, FIP1 Factor Interacting with PAP, IKK IκB Kinase, LncRNAs Long non-coding RNAs, SRSF7 Serine/Arginine-rich Splicing Factor 7, NF-kB Nuclear Factor Kappa-light-chain-enhancer of Activated B cells; NMD nonsense-mediated mRNA decay, NXF1 Nuclear RNA Export Factor 1, PCEs Pervasive Cytotoxic Exons, pPAS Polyadenylation Site, RS serine/arginine-rich, Split-ORFs Split Open Reading Frames, TREX Transcription-Export, TAP Tip-associated protein.

SRSF7, as a splicing factor, can influence the process of carcinogenesis not only by participating in mRNA splicing regulation but also by regulating nuclear export, transcription, and translation (Fig. 2).

## Molecular mechanism of SRSF7 promoting tumor progression

### SRSF7 in aberrant cell cycle regulation

Changes in genome stability are closely related to the development and progression of tumors and are considered a key factor driving carcinogenesis [42, 43]. Studies have demonstrated that abnormal regulation of the cell cycle can lead to genomic instability, thereby heightening the likelihood of aberrant cell division and carcinogenesis [44, 45]. For instance, p21, encoded by CDKN1A, inhibits the activity of cyclin-dependent kinase (CDK) through both p53-dependent and -independent pathways. This action prevents the transition from the G1 phase to the DNA synthesis phase, thereby effectively regulating the cell cycle and inhibiting excessive tumor cell proliferation [46, 47]. Saki Saijo et al. found that in colon cancer cells (HCT116), SRSF7 does not directly activate CDKN1A transcription. However, a reduction in SRSF7 expression increases the stability of CDKN1A mRNA and minimizes the degradation of p21. This, in turn, inhibits the phosphorylation of the cell cycle regulatory protein CDK2 and the tumor suppressor protein pRb, ultimately hindering the abnormal proliferation of cancer cells [48]. In a study by Fu et al. the over-expression of SRSF7 promoted the proliferation of lung cancer cells (A549, H1975, H1299, and NCM460) and colon cancer cells (HCT116). It also facilitated the skipping of exon 6 in the precursor mRNA of the cell apoptosis regulator Fas receptor, thus activating cell proliferation. The knockdown of SRSF7 promoted the inclusion of Fas exon 6, which in turn induced apoptosis in lung and colon cancer cells [13, 49].

These studies indicate that SRSF7 may influence carcinogenesis by abnormally regulating the cell cycle. However, the role of genome homeostasis in tumor progression still needs further in-depth investigation.

### SRSF7 in non-coding RNA regulation

Non-coding RNAs regulate gene expression and epigenetic states by interacting with various components of the genome [50]. Non-coding RNAs, as untranslated RNA molecules, participate in gene expression, post-transcriptional modifications, cell cycle control, and other processes [51]. Studies have shown that SRSF7 is involved in regulating various non-coding RNAs. For example, LINC01123 can specifically bind to SRSF7, and their expression is positively correlated, jointly promoting the development of colon cancer [52]. In non-small cell lung cancer (NSCLC), MALAT1 acts as a competing endogenous RNA for miR-374b-5p. By sequestering miR-374b-5p, it prevents miR-374b-5p from binding to the 3’UTR of SRSF7, thereby relieving the post-transcriptional repression of SRSF7 and increasing its expression. Correspondingly, SRSF7 overexpression can counteract the suppressive effects on cell migration and invasion caused by MALAT1 knockdown [53]. Another study demonstrated that circular RNA circTLK1 interacts with miR-876-3p, and the 5’ UTR of miR-876-3p binds to the 3’ UTR of wild-type SRSF7. As a downstream target of the circTLK1/miR-876-3p axis, SRSF7 promotes abnormal proliferation in non-small cell lung cancer. Similarly, circRNA Circ_0006006 can specifically bind to miR-924. This alleviates the inhibitory effect caused by the binding of miR-924 to the 3’UTR of SRSF7, thereby promoting the progression of NSCLC [12, 54]. In oral squamous cell carcinoma, RNA immunoprecipitation and RNA pull-down experiments confirmed that SRSF7 interacts with the long non-coding RNA (lncRNA) PANDAR as well as with the protein PIM1. After reducing PANDAR expression, SRSF7 expression was up-regulated, and PIM1 expression was down-regulated, thereby inhibiting proliferation and promoting apoptosis in oral squamous cell carcinoma [15]. In prostate cancer, the expression of miR-30e is significantly reduced. The over-expression of miR-30e induces cell cycle arrest, promotes cell apoptosis, and increases drug sensitivity. RNA immunoprecipitation experiments showed that SRSF7 acts as a downstream target of miR-30e. The suppression of miR-30e up-regulates SRSF7 expression, thereby driving abnormal cell cycle distribution in prostate cancer cells [55]. However, the precise molecular mechanisms underlying these regulatory relationships partially remain to be elucidated.

These studies suggest that the aberrant expression of SRSF7 is closely related to the initiation and progression of malignant tumors, and that SRSF7 is involved in the regulation of gene expression and protein synthesis by non-coding RNAs. However, the specific molecular mechanisms by which SRSF7 regulates the splicing, post-transcriptional modifications, translation, or protein interactions of non-coding RNA genes still require further investigation.

### SRSF7 in RNA methylation regulation

N6-methyladenosine (m6A) is the most common form of mRNA modification in eukaryotes and one of the most critical and essential RNA methylation modifications. Studies have shown that m6A-related protein methyltransferase 3 (METTL3) regulates tumor invasion by modulating the expression of methylation-related genes [56]. Research by Cun et al. reported that SRSF7, through its RRM domain, can bind to METTL3, recruiting it to the m6A sites of the SRSF7 downstream gene, PDZ-binding kinase (PBK). This forms a specific methylation pattern that promotes the proliferation, migration, and invasion of malignant glioma cells [57]. Currently, research on other SR family factors involved in RNA methylation is also limited. Further studies on the molecular mechanisms of SRSF7’s role in methylation will provide a model for studying other molecules in the SR family.

### SRSF7 in glucose metabolism reprogramming

Metabolic reprogramming in tumors is an indispensable aspect of tumor development and arises as a direct or indirect consequence of oncogenic mutations [58]. Tumor cell proliferation and growth depend on a high intake of nutrients, often necessitating metabolic reprogramming. The Warburg effect enables tumor cells to rapidly produce ATP and metabolic intermediates, supporting their rapid proliferation [59]. In hepatocellular carcinoma, the splicing factor SRSF7 promotes the expression of pyruvate kinase (PKM) by regulating the alternative splicing of PKM2, thereby driving glucose metabolic reprogramming and tumor cell proliferation in the HepG2 cell line [60].

### SRSF7 in regulating tumor immune response

#### SRSF7 promotes tumor immune activation by regulating the immune micro-environment

Immune infiltration plays a crucial role in balancing tumor development, significantly impacting various aspects of tumorigenesis, metastasis, and therapeutic interventions. As an essential member of the SRSF family, SRSF7 has garnered increasing attention for its role in modulating the tumor immune micro-environment. Zhang et al. found, through a single-cell analysis of esophageal cancer, that mast cells (MCs) with high expression of SRSF7 exhibit potential pro-cancer characteristics. Furthermore, the SRSF7 ( + ) MC score was closely associated with poor prognosis in tumors [61]. Shen et al. and Long et al., through gene enrichment and single-cell analysis, respectively, identified that in hepatic carcinoma and multiple myeloma, SRSF7 was correlated with various infiltrating inflammatory cells, including CD4^+^ T cells, monocytes/macrophages, CD8^+^ T cells, and endothelial cells [14, 16]. They also found that it was involved in several tumor immune-related signaling pathways, such as the B cell receptor signaling pathway and the mTOR signaling pathway [14]. Recent research on gastric cancer suggests that SRSF7, as an upstream splicing factor, forms a regulatory network with multiple survival-related splicing events and may modulate the tumor micro-environment by influencing the splicing patterns of immune-related genes [17]. In macrophages, SRSF7 specifically binds to exon 2 within the 5’ UTR of the nascent IRF7 transcript. By cooperating with the histone methyltransferase KMT5a, it enhances STAT1 binding at the promoter and promotes RNA polymerase II elongation, thereby activating IRF7 transcription. This mechanism reveals SRSF7 as a key regulatory node in the type I interferon signaling pathway and the immune microenvironment reorganization [62]. These studies indicate that SRSF7 influences changes in the immune microenvironment by regulating the splicing variations of key genes. Currently, most of the literature focuses on bioinformatics screening, and the specific molecular mechanisms require further exploration.

#### SRSF7 involvement in regulating PD-1/PD-L1-mediated tumor immune evasion

Immune responses primarily involve immune activation and immune evasion, and the dynamic balance between these two processes is crucial for regulating tumor initiation and progression. It also forms the essential foundation for tumor immunotherapy. Programmed cell death protein 1 (PD-1) is an immune checkpoint receptor mainly located on the surface of immune cells such as T cells, B cells, and dendritic cells. By binding to its ligand, programmed death-ligand 1 (PD-L1), PD-1 inhibits immune cells’ function and induces apoptosis, thereby impacting tumor immunotherapy and facilitating immune evasion by cancer cells [63, 64]. Recent research indicates SRSF7, part of the SRSF family, is crucial in tumor immune evasion. Bio-informatics enrichment analysis results have shown that SRSF7 is correlated with TNF-α, which can up-regulate PD-L1 expression by activating NF-kB, promoting immune evasion and metastasis [16, 65, 66]. Therefore, it is speculated that SRSF7 may participate in regulating tumor immune responses through TNF-α.

Research by Mo et al. indicated that cancer-associated fibroblasts (CAFs) with high SRSF7 expression serve as biomarkers for gastric cancer prognosis and immune checkpoint blockade [67]. Studies on non-coding RNAs and SRSF7 showed a dual-luciferase reporter assay revealed a binding site between the 3’ UTR of SRSF7 and the 5’ UTR of miR-876-3p. This interaction indirectly regulates the expression of circTLK1, thereby being involved in regulating tumor immune evasion [54]. Lai et al. found that infection of cells with a BTV reporter gene containing the PD-1 3’ UTR region revealed that SRSF7 binds to the PD-1 3’ UTR region. Following CRISPR-Cas9-mediated knockout of SRSF7, PD-1 protein levels were only slightly affected. SRSF7, along with other RNA-binding proteins (RBPs) such as IGF2BP2 and RBM38, may suppress PD-1 expression by influencing mRNA stability, which significantly inhibits the expression of IL-2 and CD4 in MOLT-4 cells from acute lymphoblastic leukemia [68].

Given the expression of SRSF7 in immune infiltration and the roles of SRSF family members in tumor immune activation and immune evasion, it is important to further explore how SRSF7 regulates tumor immune responses at the molecular level.

**Fig. 3:**
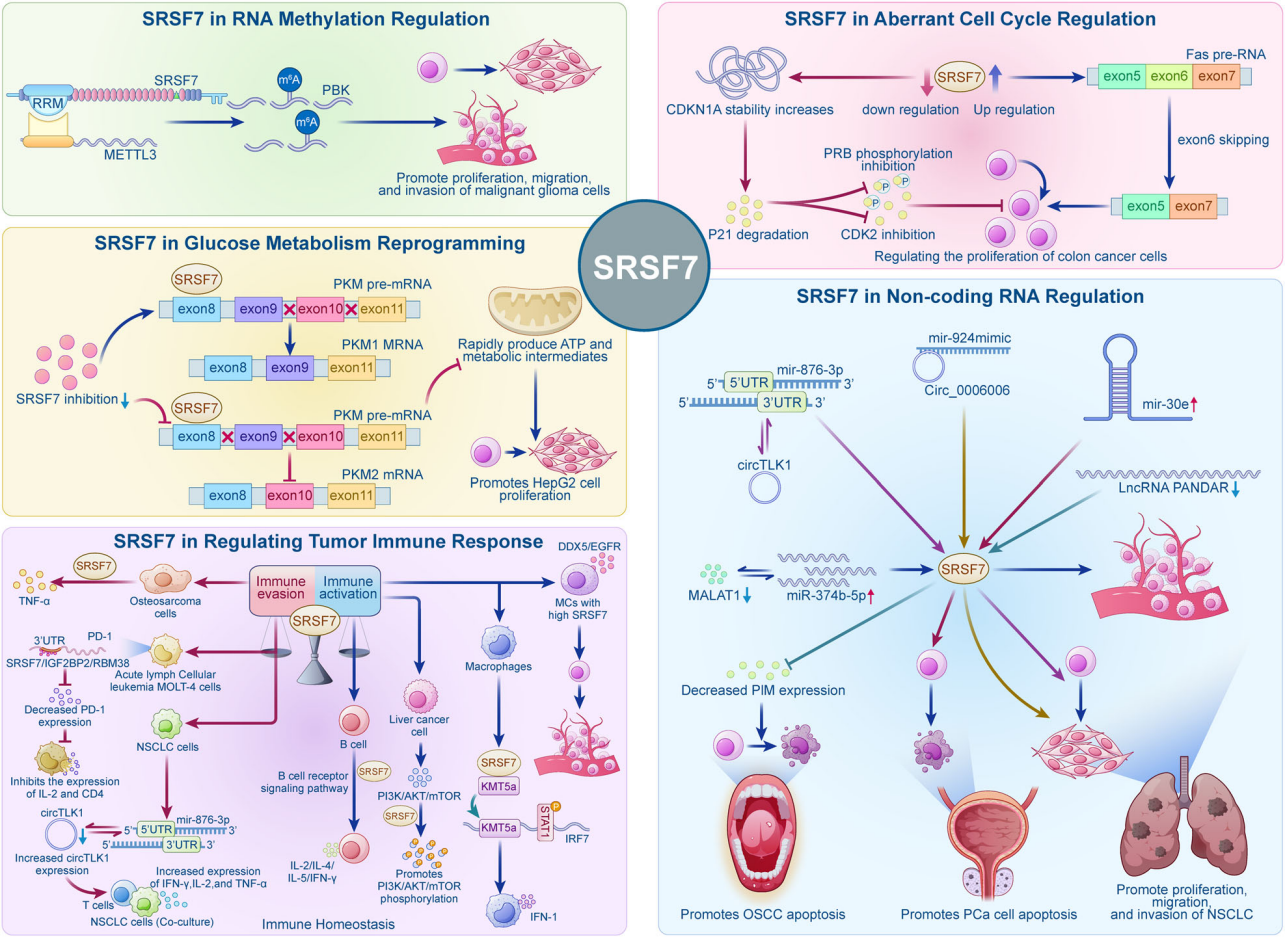
Molecular mechanisms of SRSF7 in driving tumor progression and modulating tumor immune responses. SRSF7 mediates tumor proliferation/apoptosis by regulating CDKN1A stability and Fas pre-mRNA splicing; SRSF7 orchestrates a complex ceRNA network by collaborating with multiple non-coding RNAs, including LINC01123, MALAT1, circTLK1, and miR-876-3p, and triggers tumor proliferation, invasion, and progression in various cancers, including CRC and NSCLC; SRSF7 recruits METTL3 to install PBK-specific m⁶A modifications driving glioblastoma progression; SRSF7 reprograms PKM isoform splicing to promote metabolic proliferation in hepatocellular carcinoma; SRSF7 remodels the tumor microenvironment by targeting IRF7-related immune pathways in tumor/immune cells, while facilitating PD-1/PD-L1-mediated immune evasion. 3’ UTR 3’ Untranslated Region, 5’ UTR 5’ Untranslated Region, ATP Adenosine Triphosphat, CD4 Cluster of Differentiation 4, CDK Cyclin-Dependent Kinase, CDKN1A Cyclin-Dependent Kinase Inhibitor 1A, CRC Colorectal Cancer, DDX5 DEAD-Box Helicase 5, EGFR Epidermal Growth Factor Receptor, IL interleukin, IFN-γ interferon-gamma, IRF7 Interferon Regulatory Factor 7, MALAT1 Metastasis-Associated Lung Adenocarcinoma Transcript 1, METTL3 methyltransferase 3, mTOR mammalian Target of Rapamycin, m⁶A N6-methyladenosine, NSCLC non-small Cell Lung Cancer, OSCC Oral Squamous Cell Carcinoma, PD-1 Programmed Cell Death Protein 1, PD-L1 programmed death-ligand 1, PI3K Phosphatidylinositol 3-Kinase, PKM Pyruvate Kinase Muscle, PCa Prostate Cancer, PRB Retinoblastoma Protein; RRM RNA Recognition Motif, SRSF7 Serine/Arginine-rich Splicing Factor 7, STAT1 Signal Transducer and Activator of Transcription 1, TNF-α Tumor Necrosis Factor-alpha.

In conclusion, SRSF7 is not only involved in regulating tumor cell cycles and non-coding RNAs, but also in regulating RNA methylation and glucose metabolism in tumor cells. Within the tumor micro-environment, SRSF7 can induce immune cell infiltration to regulate tumor immune responses and also regulate PD-1-mediated immune evasion (Fig. 3).

## Research breakthroughs and clinical translational potential of SRSF7 in tumor therapy

In traditional tumor therapies, drug resistance remains one of the most prevalent and significant challenges. Whether it is chemotherapy, targeted therapy, or immunotherapy, drug resistance reduces the effectiveness of treatment, leading to further disease progression. The primary mechanisms often focus on tumor cells mutating or altering metabolic pathways to diminish drug absorption or augment drug elimination [69]. The other members of the SRSF family primarily participate in tumor drug resistance through classic or non-classic splicing mechanisms (Table 1).

**Table 1:** Tumor drug resistance processes involving the other members of the SRSF family.

Name	Tumor Type	Molecular mechanisms involved in drug resistance	Reference
SRSF1	Bladder Cancer	SRSF1 interacts with pre-HIF1A, enhances HIF1 expression through alternative splicing, and activates the HIF1A/BNIP3 pathway. This process promotes mitochondrial autophagy in tumor cells, thereby reducing sensitivity to Cisplatin.	[71]
	Breast Cancer	The active ingredient Cyperotundone in the traditional herb Cyperus suppresses SRSF1 expression in resistant tumor cells, and regulates the alternative splicing of MYO1B to enhance resistance to Adriamycin.	[72]
	Triple-Negative Breast Cancer	SRSF1 up-regulates the circSEPT9/GCH1 axis to inhibit ferroptosis, thereby decreasing the sensitivity of triple-negative breast cancer cells to Cisplatin.	[73]
	Colon Cancer	FAM135B mediates resistance to Oxaliplatin in colorectal cancer by promoting the alternative splicing of FAAP20, a process driven by SRSF1.	[74]
	Non-Small-Cell Lung Cancer	Circ_0001786 targets miR-34b-5p to induce SRSF1 expression and enhances resistance to Gefitinib by promoting malignant cell behaviors.	[75]
	Non-Small-Cell Lung Cancer	The up-regulation or abnormal phosphorylation of SRSF1 enhances the splicing of caspase 9 pre-mRNA, generating the anti-apoptotic splice variant caspase 9b. This process inhibits apoptosis and causes resistance to chemotherapeutic drugs (Daunorubicin, Cisplatin, Paclitaxel).	[76]
	Refractory Acute Myeloid Leukemia	Overexpression of SRSF1 promotes the expression of the BCL2L11 γ splice variant, thereby inhibiting apoptosis and inducing resistance against Omipalisib.	[77]
	Ovarian Cancer	CRNDE activates the SRSF1/TIA1 pathway to further enhance ovarian cancer’s resistance to Cisplatin.	[78]
	Glioma	SRSF1 interacts with LINC01564, stabilizing its mRNA, which in turn increases NFE2L2 expression, leading to apoptosis and ferroptosis and promoting the resistance of glioma cells to Cisplatin.	[79]
	Chronic Myelogenous Leukemia	Elevated SRSF1 levels mediate the up-regulation of PRKCH and PLCH1 through gene expression and alternative splicing, reducing sensitivity to Imatinib.	[80]
SRSF2	Prostate Cancer	SRSF2 mediates exon 20 skipping in PIK3CD pre-mRNA, generating the PI3Kδ-S variant with lower drug affinity, promoting resistance to PI3Kδ inhibitors.	[81]
	Gastric Cancer	MicroRNA-193a-3p expression increases in CD44(+) gastric cancer cells, suppressing SRSF2 expression and weakening its splicing function. This leading to decreased expression of pro-apoptotic genes (e.g., Bax, cytochrome C, cleaved caspase 3, and caspase 9), while anti-apoptotic genes (Bcl-2) are up-regulated, increasing resistance to Cisplatin.	[82]
	Ovarian Cancer	SRSF2 competes with hnRNPA1 to bind to AUF1 pre-mRNA, regulating its splicing to generate subtypes that affect ovarian cancer cell sensitivity to Cisplatin, thereby playing a key role in Cisplatin resistance.	[83]
	Ovarian Cancer	SFPQ and p54NRB form a complex that inhibits SRSF2 activity, thereby affecting its alternative splicing of caspase-9. This promotes the expression of anti-apoptotic splice variants, leading to resistance to platinum chemotherapy.	[84]
	Ovarian Cancer	LncRNA PANDAR interacts with SRSF2, inhibiting p53 Ser15 phosphorylation, which reduces p53-mediated apoptosis gene expression and decreases sensitivity to Cisplatin, leading to ovarian cancer resistance.	[85]
	Bladder Cancer	MiR-193a-3p targets and suppresses the expression of SRSF2, PLAU, and HIC2, potentially through multiple signaling pathways such as oxidative stress, Notch, and DNA damage, thereby increasing bladder cancer’s resistance to multiple chemotherapy drugs like Pirarubicin, Paclitaxel, Doxorubicin, and Epirubicin Hydrochloride.	[86–88]
	Liver Cancer	MiR-193a-3p targets and suppresses SRSF2, regulating the splicing of apoptosis-related genes and promoting resistance to 5-fluorouracil in hepatocellular carcinoma.	[89]
SRSF3	Pancreatic Cancer	SRSF3 promotes gemcitabine resistance in pancreatic cancer by regulating the splicing of ANRIL, providing a crucial and novel mechanism beyond widely known ones such as ARNTL2 and cFAM124A in the regulation of PI3K/Akt signaling pathway.	[90–92]
SRSF4	Glioma	SRSF4 positively regulates MDC1 expression through alternative splicing, enhancing MDC1’s role in promoting resistance to Temozolomide by accelerating DNA double-strand break repair and lowering glioma cell drug sensitivity.	[93]
SRSF5	Colon Cancer	LINC01852 inhibits SRSF5-mediated PKM alternative splicing, reducing the production of PKM2 associated with aerobic glycolysis. This mechanism inhibits colorectal cancer growth and enhances sensitivity to chemotherapy drugs like 5-fluorouracil and Oxaliplatin.	[94]
SRSF6	Gastric Cancer	The long non-coding RNA RCNRNDE binds to SRSF6, resulting in decreased protein stability and alterations in PICALM splicing. By controlling the skipping of PICALM exon 14 variants, PICALM expression is raised, autophagic activity is boosted, and resistance to 5-fluorouracil and Oxaliplatin is promoted.	[95]
	Thyroid Cancer	SRSF6 regulates alternative splicing via NSUN2-mediated m5C modification, which promotes N-glycosylation and stability of ABC transporters, thereby enhancing thyroid cancer’s resistance to Doxorubicin, Cisplatin, and Lenvatinib.	[96]
SRSF9	Ovarian Cancer	SRSF9 promotes the recruitment of PANDAR to nucleosomes, inhibiting apoptosis and increasing resistance to Cisplatin.	[97]

In recent years, studies have shown that miR-30e interacts with SRSF7 and regulates its expression, consequently increasing the cytotoxic effects of chemotherapy drugs such as Enzalutamide and Abiraterone on androgen receptor (AR)-positive castration-resistant prostate cancer (CRPC) cells, thereby increasing drug sensitivity [55]. In dormant and reactivated lung cancer A549 cells, alternative splicing events involving SRSF7 are generally suppressed, which may be associated with changes in the expression of splicing factors, further regulating the expression of genes related to the cell cycle and DNA repair [70]. Splicing factors, RNA helicases, and alternative splicing play intricate roles in the adaptive response of A549 lung cancer cells to chemotherapy stress. They are involved in regulating cell dormancy and reactivation, providing new therapeutic strategies for combating chemotherapy resistance and tumor recurrence [70].

However, as an important member of the SRSF family, although SRSF7 can regulate the drug sensitivity of certain tumor cells, the scope and depth of research are still limited, and many issues still need further in-depth exploration.

## Challenges and future directions

This review examines the pro-oncogenic function of SRSF7 in various tumors, including lung and prostate cancers, where it facilitates tumor development by enhancing cell proliferation and invasion [54, 55]. These findings highlight the diversity and variability of SRSF7’s functions. Furthermore, bioinformatic analyses combined with experimental evidence suggest that SRSF7 participates in key pathways, such as PD-1 and IRF7 [62, 68]. Fu et al. confirmed that SRSF7 was critical for the survival of colon and lung cancer cells [13], and Shen et al. found that SRSF7 was highly expressed in HCC patients and was associated with a poor prognosis [14]. Although SRSF7 has been proposed as a diagnostic and prognostic biomarker among various cancers, larger-scale experimental and clinical studies are necessary to confirm its clinical application value. Whether the anti-tumor potential of SRSF7 can be achieved through small-molecule inhibitors or gene editing strategies still needs thorough evaluation of its reliability, safety, targeting ability, and risk of drug resistance. The aim to develop SRSF7-specific inhibitors based on solid fundamental research, utilizing its splicing regulatory network and exploring its synergistic effects with immune checkpoint inhibitors will provide new strategies for cancer treatment.

In conclusion, as a core regulator of RNA splicing, the study of SRSF7 enhances our molecular understanding of tumorigenesis and provides new opportunities for targeted interventions in precision medicine. Through interdisciplinary collaboration and technological innovation, SRSF7 is expected to become an essential target for cancer therapies and companion diagnostics, promoting significant development from basic research to clinical translation.

## Data Availability

No data and materials were used for the research described in the article.
